# Supplementary material for: Menin directs regionalized decidual transformation through epigenetically setting PTX3 to balance FGF and BMP signaling
Source: Nat Commun. 2022 Feb 22;13:1006. doi: 10.1038/s41467-022-28657-2 (PMC8864016; doi:10.1038/s41467-022-28657-2)
Supplement: Supplementary file 1 — Supplementary Information [file 41467_2022_28657_MOESM1_ESM.pdf]

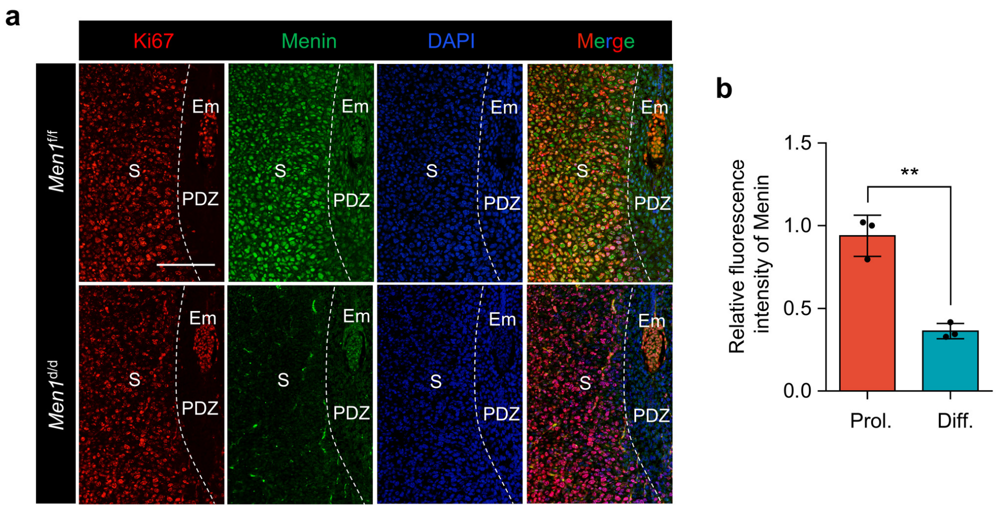

Supplementary Fig. 1 Menin is more intensely expressed in proliferating stromal cells than that in differentiated cells. (a) Immunofluorescence colocalization analysis of Ki67 and Menin in *Men1*$^{f/f}$ and *Men1*$^{d/d}$ implantation sites on day 6. Scale bar: 200 μm. **(b)** Quantitative analysis of relative fluorescence intensity of Menin in proliferating stromal cells (Prol.) and differentiated cells (Diff.). Data represent the mean ± SEM (n = 3 biologically independent samples). Two-tailed unpaired Student's t test, ***p*=0.0017.

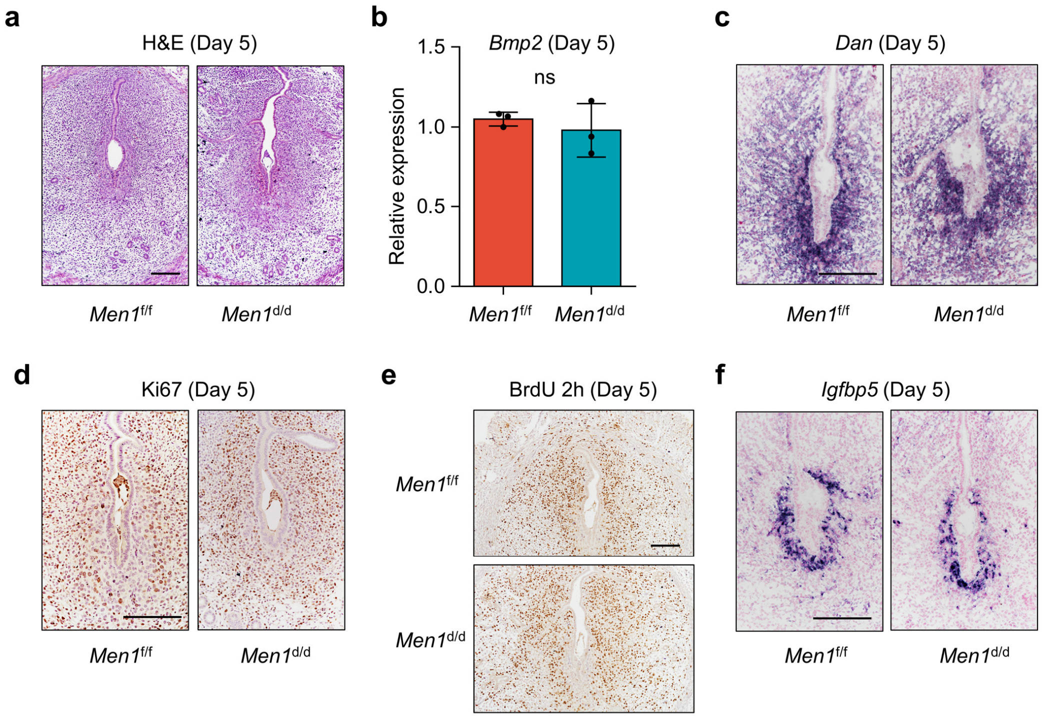

Supplementary Fig. 2 Attachment reaction of blastocyst is comparable between *Men1*$^{f/f}$ and *Men1*$^{d/d}$ females in day 5 uteri. (a) Histology of day 5 implantation sites. Scale bar: 200 μm. **(b)** Quantitative real-time PCR analysis of *Bmp2* in *Men1*$^{f/f}$ and *Men1*$^{d/d}$ implantation sites on day 5. The values are normalized to *Gapdh* and indicated as the mean ± SEM (n = 3 biologically independent samples). Two-tailed unpaired Student's t test. ns, not significant. **(c)** In situ hybridization of *Dan* in *Men1*$^{f/f}$ and *Men1*$^{d/d}$ uteri on day 5. Scale bar: 200 μm. **(d, e)** Immunohistochemistry of Ki67 and BrdU in *Men1*$^{f/f}$ and *Men1*$^{d/d}$ implantation sites on day 5. Scale bar: 200 μm. **(f)** In situ hybridization of *Igfbp5* in *Men1*$^{f/f}$ and *Men1*$^{d/d}$ implantation sites on day 5. Scale bar: 200 μm.

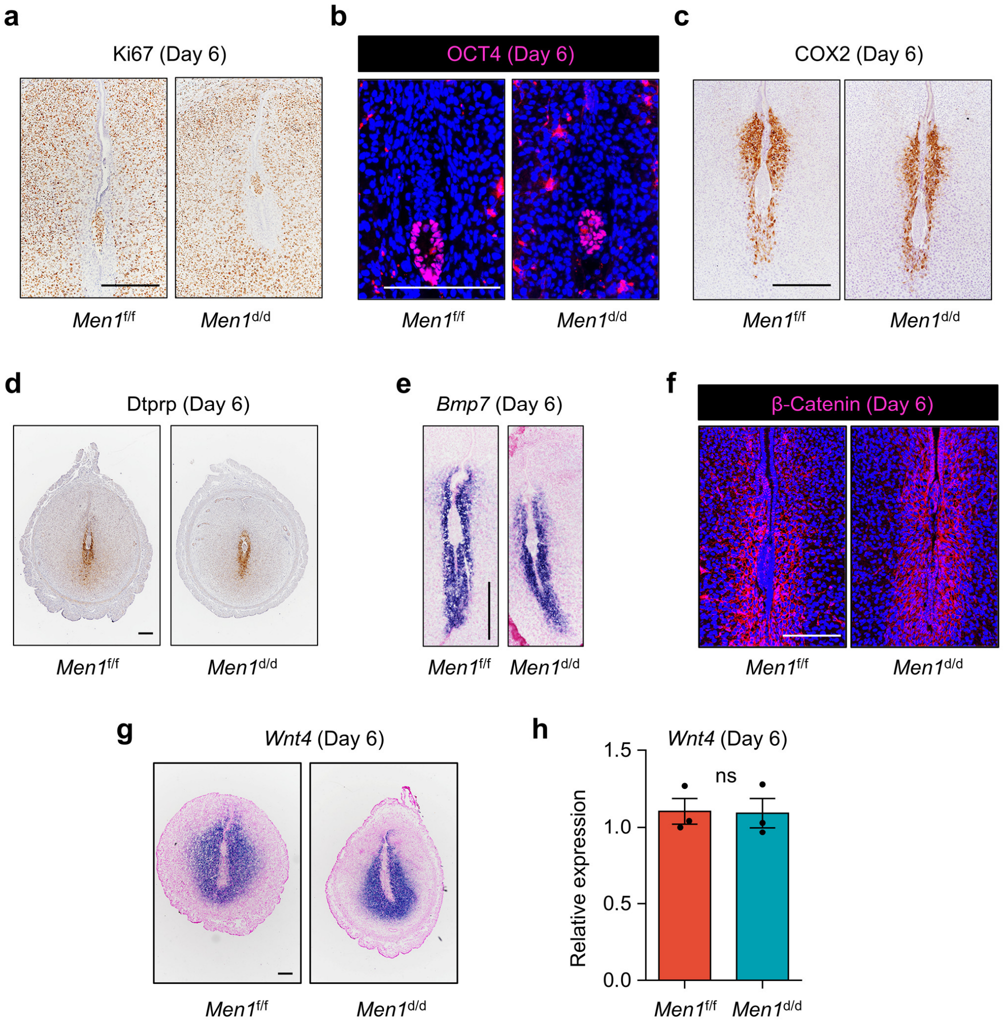

Supplementary Fig. 3 Initiation of decidualization is comparable between *Men1*$^{f/f}$ and *Men1*$^{d/d}$ uteri on day 6. (a) Immunohistochemistry of Ki67 in *Men1*$^{f/f}$ and *Men1*$^{d/d}$ implantation sites on day 6. Scale bar: 200 μm. Similar results were obtained in at least three independent experiments. **(b)** Immunofluorescence analysis of OCT4 in *Men1*$^{f/f}$ and *Men1*$^{d/d}$ implantation sites on day 6. Scale bar: 200 μm. Similar results were obtained in at least three independent experiments. **(c, d)** Immunohistochemistry of COX2 and Dtprp in *Men1*$^{f/f}$ and *Men1*$^{d/d}$ implantation sites on day 6. Scale bar: 200 μm. Similar results were obtained in at least three independent experiments. **(e)** In situ

hybridization of *Bmp7* in *Men1*$^{f/f}$ and *Men1*$^{d/d}$ implantation sites on day 6. Scale bar: 200 μm. Similar results were obtained in at least three independent experiments. **(f)** Immunofluorescence analysis of β-Catenin in *Men1*$^{f/f}$ and *Men1*$^{d/d}$ implantation sites on day 6. Scale bar: 200 μm. Similar results were obtained in at least three independent experiments. **(g)** In situ hybridization of *Wnt4* in *Men1*$^{f/f}$ and *Men1*$^{d/d}$ implantation sites on day 6. Scale bar: 200 μm. **(h)** Quantitative real-time PCR analysis of *Wnt4* in *Men1*$^{f/f}$ and *Men1*$^{d/d}$ implantation sites on day 6. The values are normalized to *Gapdh* and indicated as the mean ± SEM (n = 3 biologically independent samples). Two-tailed unpaired Student's t test. ns, not significant.

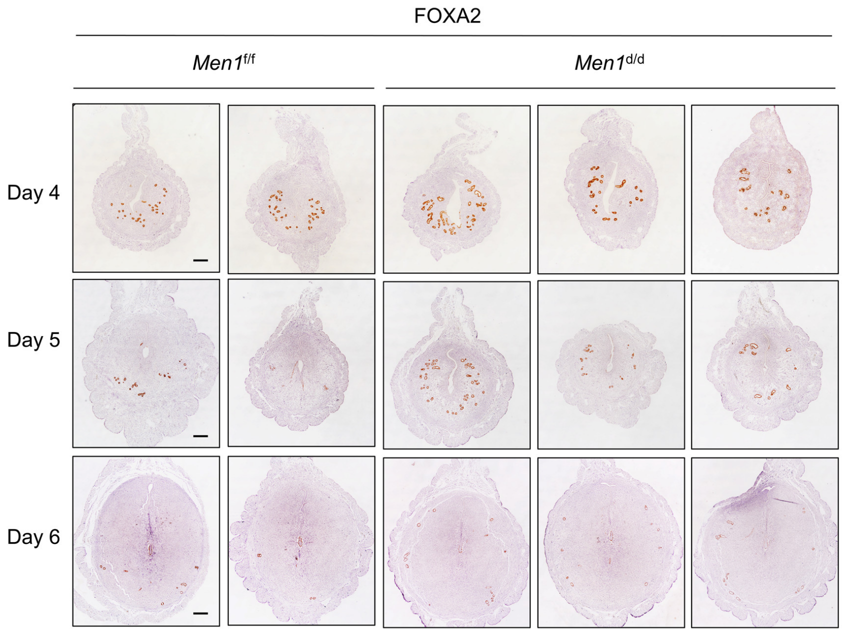

Supplementary Fig. 4 Increased number of glands in the lateral areas near the M pole in stroma of *Men1*$^{d/d}$ uteri. Immunohistochemistry of FOXA2 in *Men1*$^{f/f}$ and *Men1*$^{d/d}$ uteri on pregnant days 4,5 and 6. Scale bar: 200 μm. Similar results were obtained in at least three independent experiments.

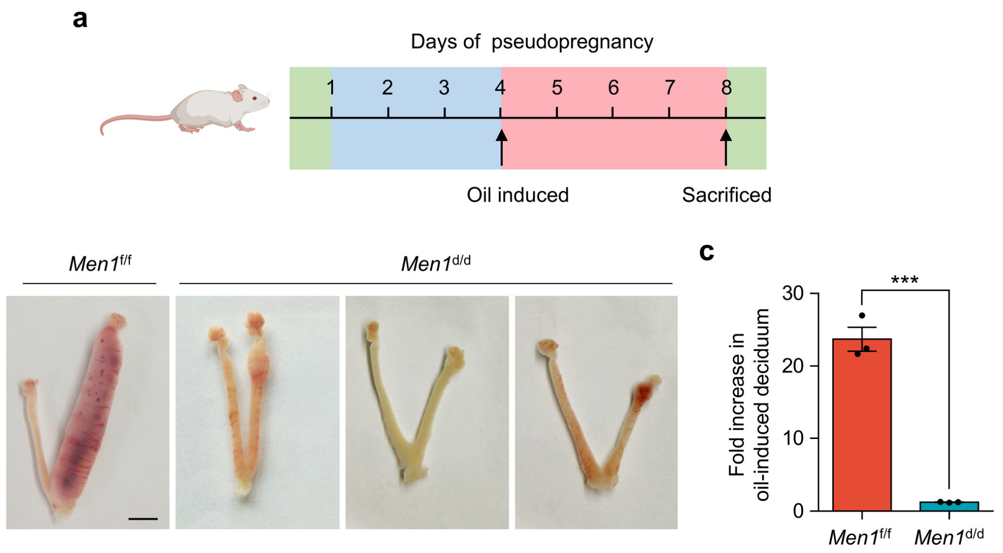

Supplementary Fig. 5 Loss of *Men1* impairs uterine decidualization in oil-induced model. (a) Schematic diagram of surgical regimen used to induce decidual response. Mice were treated with oil (25 μl) in one uterine horn on day 4 of pseudopregnancy. Uteri were collected on day 8. The icon of mouse was created with BioRender.com. **(b)** Gross morphology of the decidual response in *Men1*$^{f/f}$ and *Men1*$^{d/d}$ uteri on day 8 of pseudopregnancy. Scale bar: 5 mm. **(c)** Ratio of the decidual horn to the control horn weight of *Men1*$^{f/f}$ and *Men1*$^{d/d}$ uteri on day 8 of pseudopregnancy. Data represent the mean ± SEM (n = 3 biologically independent samples). Two-tailed unpaired Student's t test, ***p=0.0002.

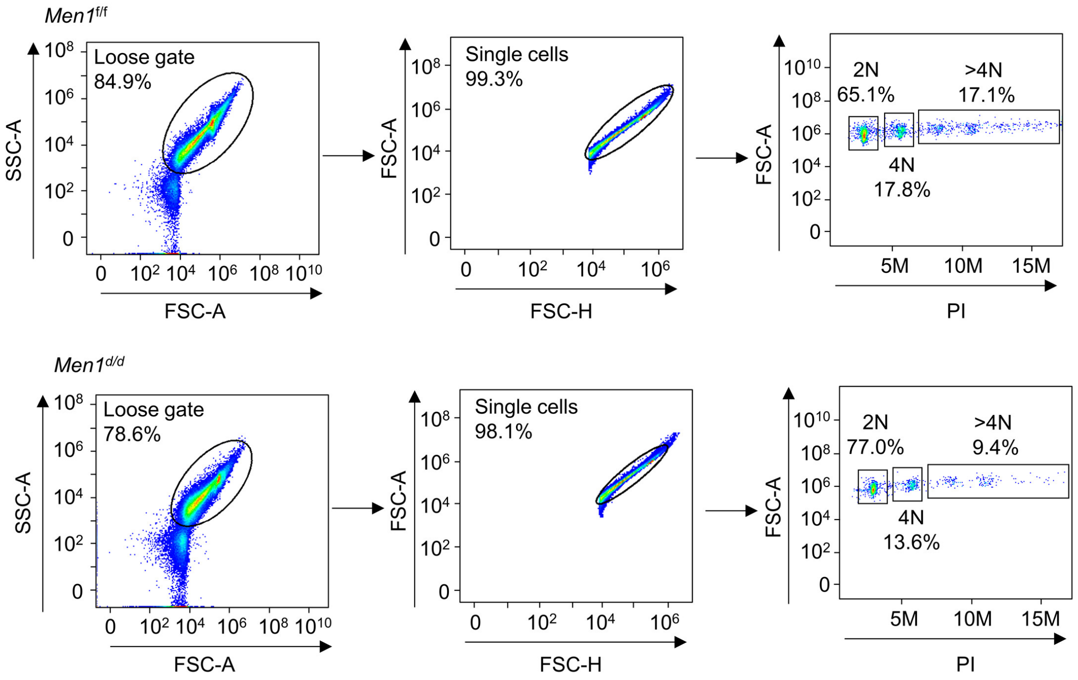

Supplementary Fig. 6 Gating strategy of DNA content for single cell. The intensity value of SSC-A and FSC-A of fluorescence activated cell sorting (FACS) are used for loose gate. The intensity value of FSC-A and FSC-H of FACS are used to gate single cells. The polyploid cells are distinguished by intensity value of FSC-A which is proportional to cell size and the intensity value of PI staining which is used to measure DNA content. PI, Propidium Iodide.

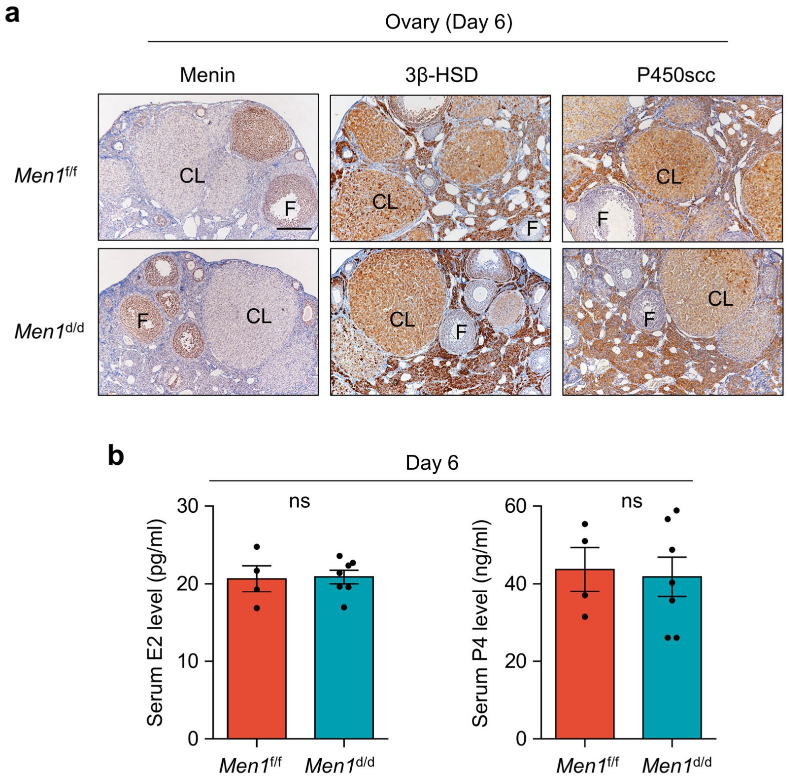

Supplementary Fig. 7 Ovarian steroid hormone secretion is comparable between *Men1*$^{f/f}$ and *Men1*$^{d/d}$ mice. (a) Immunohistochemistry staining of Menin, 3β-HSD and P450scc in day 6 *Men1*$^{f/f}$ and *Men1*$^{d/d}$ ovaries. Scale bar: 200μm. CL, corpus luteum; F, follicle. **(b)** Serum estradiol-17β (E2) and progesterone (P4) levels in *Men1*$^{f/f}$ (n = 4 animals) and *Men1*$^{d/d}$ (n = 7 animals) mice on day 6. Data represent the mean ± SEM. Two-tailed unpaired Student's t test. ns, not significant.

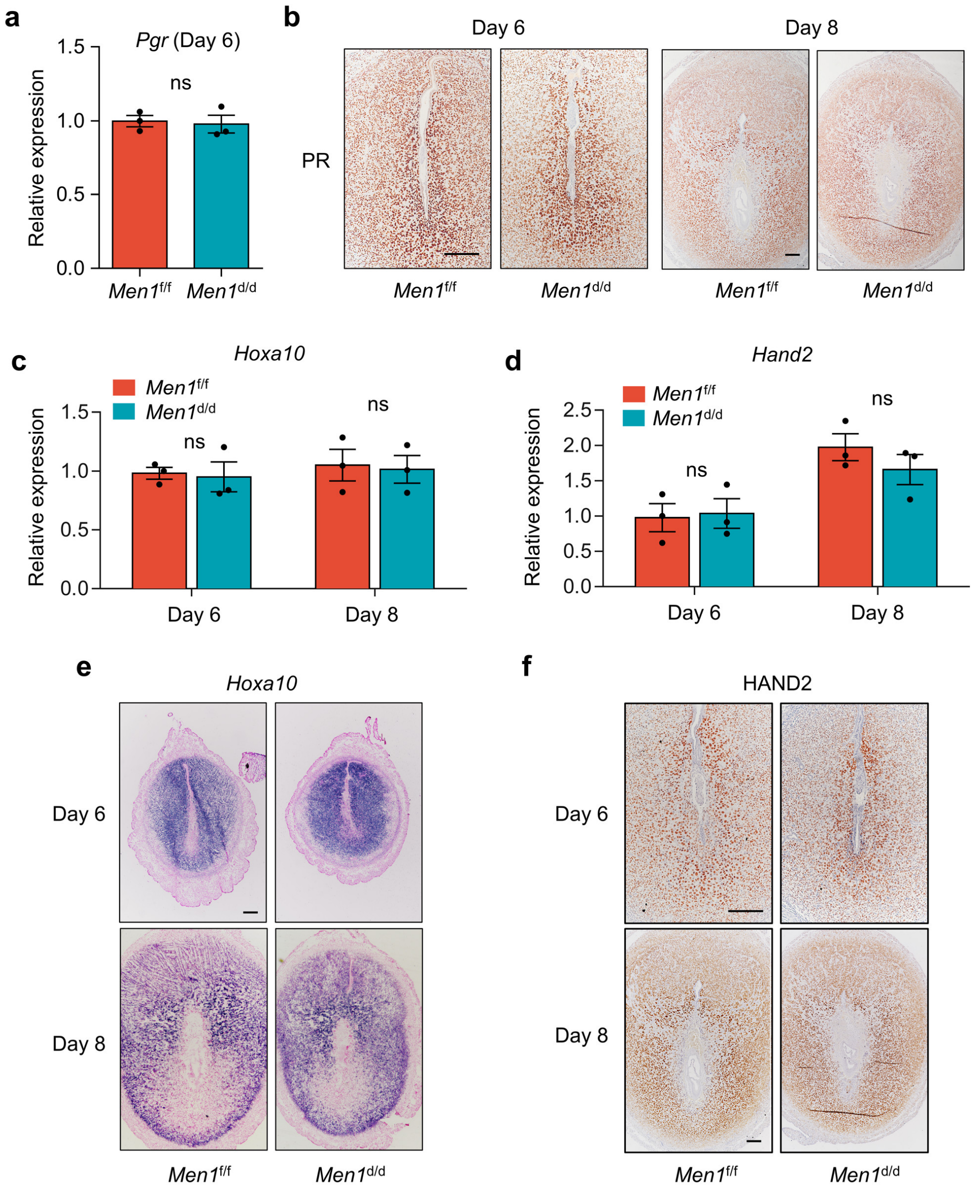

Supplementary Fig. 8 Uterine deficiency of *Men1* has no adverse effect on progesterone signaling pathway. (a) Quantitative real-time PCR analysis of *Pgr* in *Men1*$^{f/f}$ and *Men1*$^{d/d}$ uteri on day 6. The values are normalized to *Gapdh* and indicated as the mean ± SEM (n = 3 biologically independent samples). Two-tailed unpaired Student's t test. ns, not significant. **(b)** Immunohistochemistry of PR in the implantation sites of *Men1*$^{f/f}$ and *Men1*$^{d/d}$ mice on day 6 and day 8 of pregnancy. Scale bars: 200 μm. **(c, d)** Quantitative

real-time PCR analysis of *Hand2* and *Hoxa10* in *Men1*$^{f/f}$ and *Men1*$^{d/d}$ uteri on day 6 and day 8. The values are normalized to *Gapdh* and indicated as the mean ± SEM (n = 3 biologically independent samples). Two-tailed unpaired Student's t test. ns, not significant. **(e)** In situ hybridization of *Hoxa10* in *Men1*$^{f/f}$ and *Men1*$^{d/d}$ implantation sites on day 6 and day 8. Scale bar: 200 μm. **(f)** Immunohistochemistry of HAND2 in *Men1*$^{f/f}$ and *Men1*$^{d/d}$ implantation sites on day 6 and day 8. Scale bars: 200 μm.

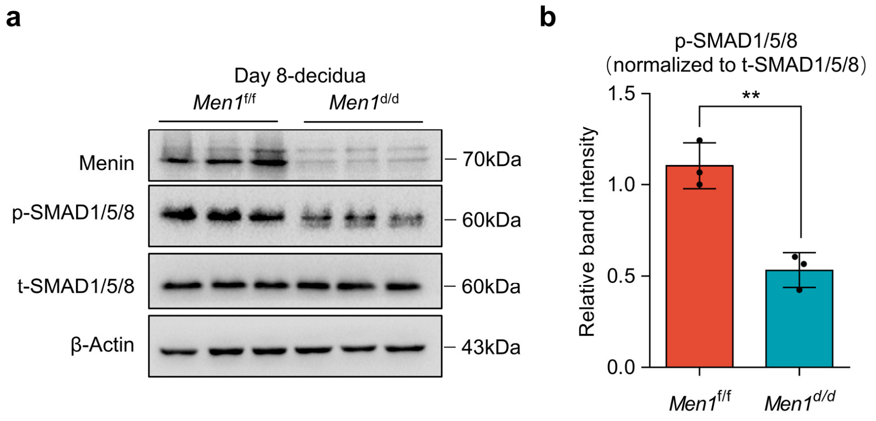

Supplementary Fig. 9 Phosphorylated SMAD1/5/8 was decreased in *Men1*$^{d/d}$ decidual cells. (a) Immunoblotting analysis of p- SMAD1/5/8 in *Men1*$^{f/f}$ and *Men1*$^{d/d}$ uteri. B-Actin and t-SMAD1/5/8 were used as loading controls. **(b)** Quantitative analysis of p-SMAD1/5/8 band intensity normalized to t-SMAD1/5/8. Data represent the mean ± SEM (n = 3 biologically independent samples). Two-tailed unpaired Student's t test, ***p*=0.0033.

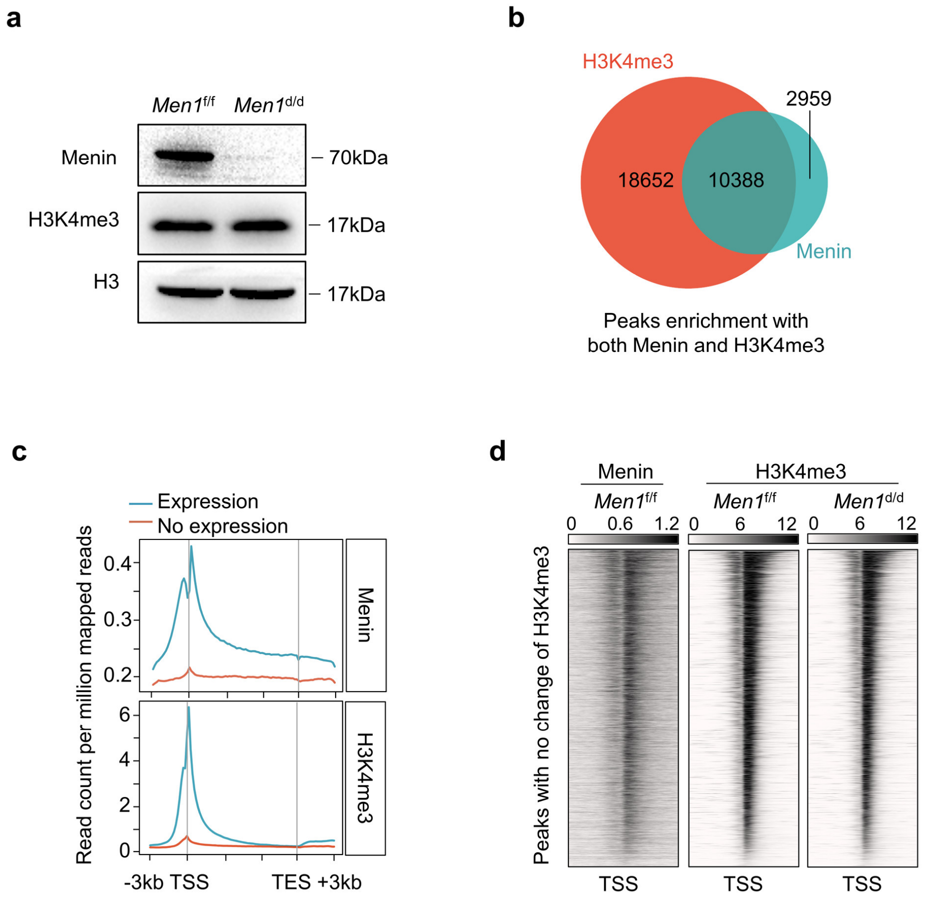

Supplementary Fig. 10 Uterine specific deletion of *Men1* does not affect the global H3K4me3 level. (a) Immunoblotting analysis of H3K4me3 in *Men1*$^{f/f}$ and *Men1*$^{d/d}$ decidual tissues on day 8. H3 was used as loading control. Similar results were obtained in at least three independent experiments. **(b)** Venn diagram of peaks enriched by H3K4me3 and Menin. **(c)** Menin and H3K4me3 ChIP-seq signal density in the genome. **(d)** Coverage profiles for Menin and H3K4me3. Heatmap of peaks with no changed H3K4me3 following *Men1* deletion centered at TSS. TSS, transcriptional start site.

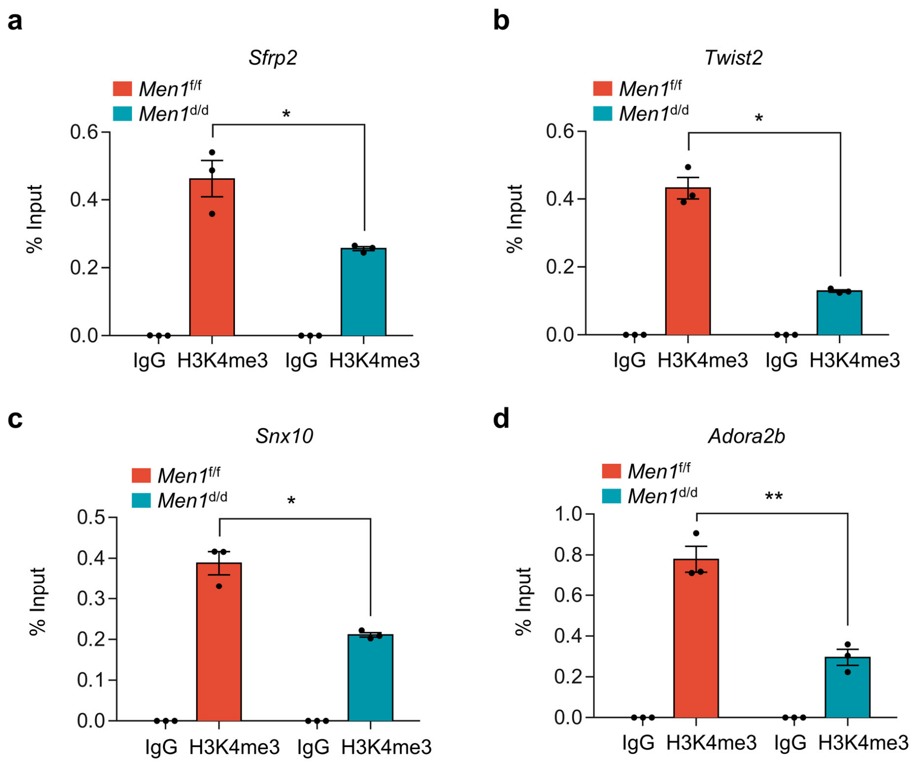

Supplementary Fig. 11 The promoter of Menin target genes have a reduced H3K4me3 modification upon uterine *Men1* deletion. (a-d) Quantitative ChIP analysis of H3K4me3 in promoter of Menin-H3K4me3 target genes in *Men1*$^{f/f}$ and *Men1*$^{d/d}$ uterine decidual tissue. Data represent the mean ± SEM (n = 3 biologically independent samples). Two-tailed unpaired Student's t test, *p=0.019 (*Sfrp2*), *p=0.0103 (*Twist2*), *p=0.022 (*Snx10*), **p=0.003 (*Adora2b*).

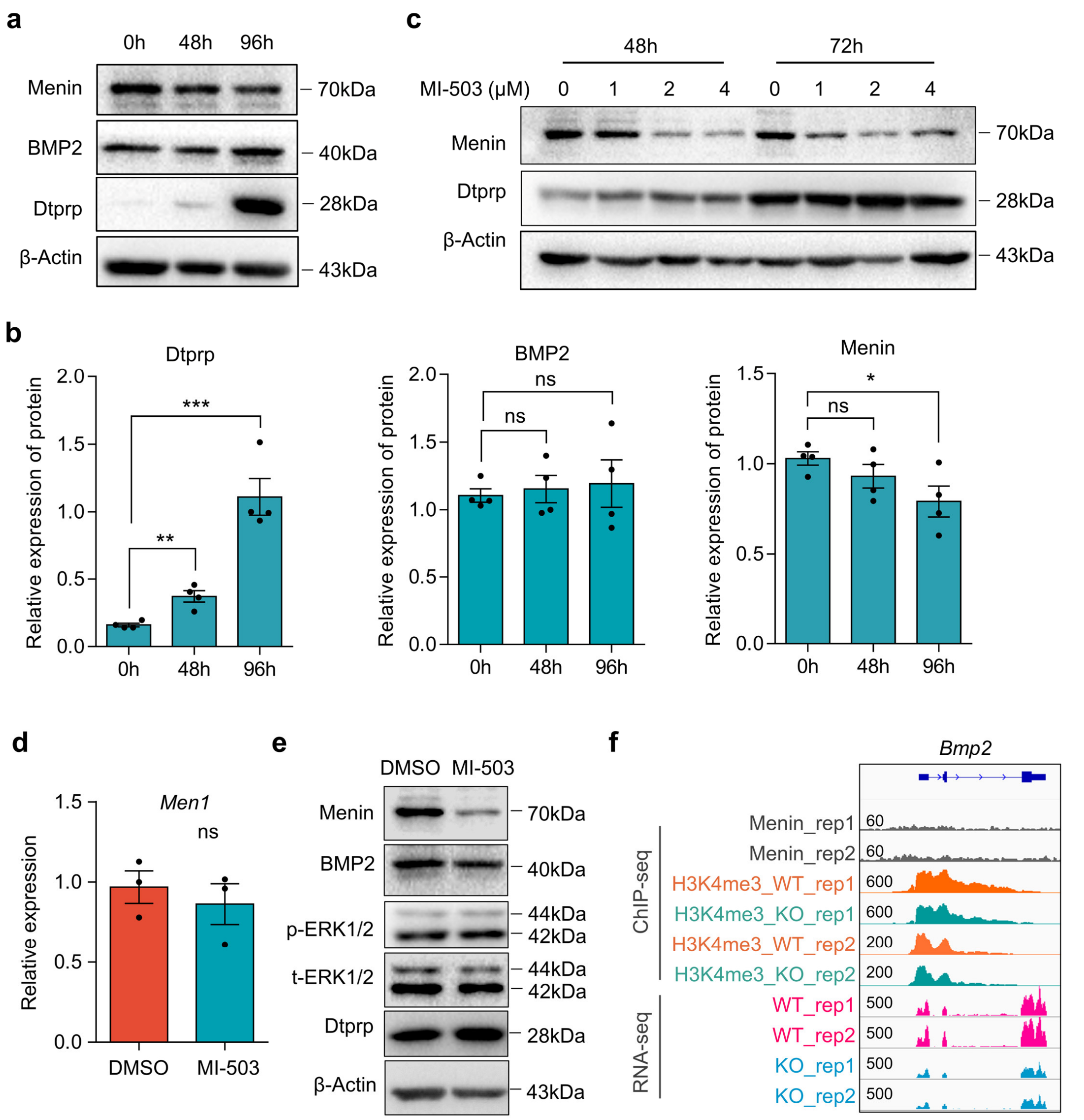

Supplementary Fig. 12 MI-503 Treatment has no effect on the expression of Bmp2 in the absence of Fgf2. (a) Immunoblotting analysis of Menin, BMP2 and Dtprp in mESC during in vitro decidualization. B-Actin was used as loading control. **(b)** Quantitative analysis of Dtprp, BMP2 and Menin band intensity normalized to β-Actin during in vitro decidualization. Data represent the mean ± SEM (n = 4 biologically independent samples). Two-tailed unpaired Student's t test, ***p*=0.003, ****p*=0.0004, **p*=0.0434, ns, not significant. **(c)** Immunoblotting analysis of Menin expression in the presence of 0, 1, 2, or 4 μM

MI-503 in mESC. B-Actin was used as loading control. **(d)** Quantitative real-time PCR analysis of *Men1* in mESC during in vitro decidualization with or without treatment of 2 μM MI-503 for 72h. The values are normalized to *Gapdh* and indicated as the mean ± SEM (n = 3 biologically independent samples). Two-tailed unpaired Student's t test. ns, not significant. **(e)** Immunoblotting analysis of Menin, BMP2 and p-ERK1/2 in mESC treatment with MI-503 (2 μM) for 72h during in vitro decidualization. Total ERK1/2 and β-Actin were used as loading controls. **(f)** Genome browser view of normalized ChIP-seq signals of Menin and H3K4me3 and RNA-seq tracks for *Bmp2* in *Men1*$^{f/f}$ and *Men1*$^{d/d}$ decidual tissues.

Fig.1d

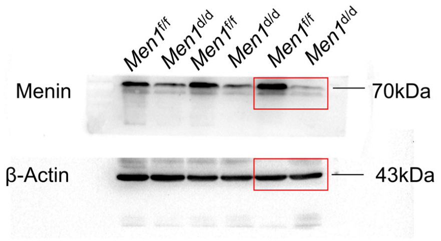

Fig.3f

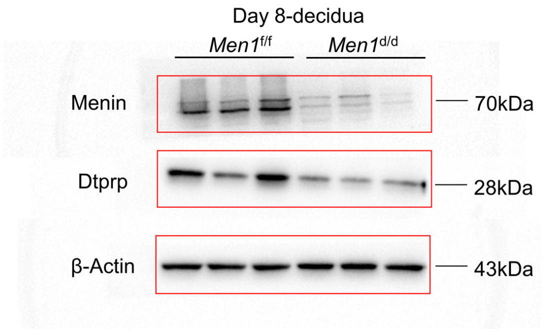

Fig.5e

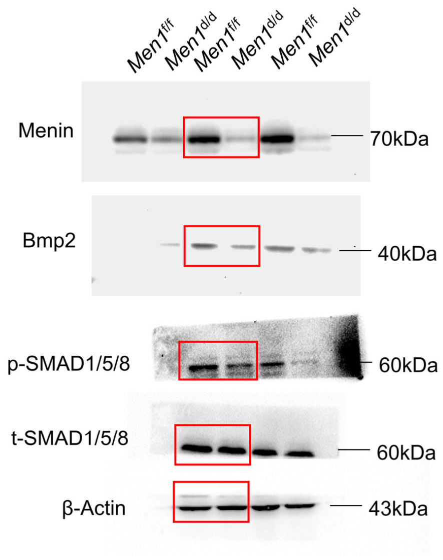

Fig.7f

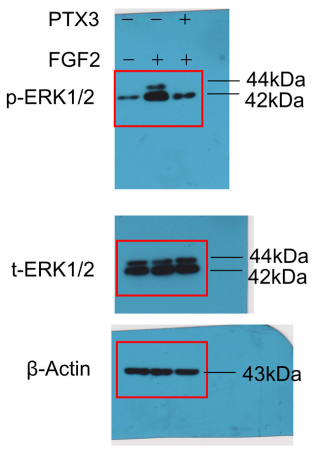

Fig.7h

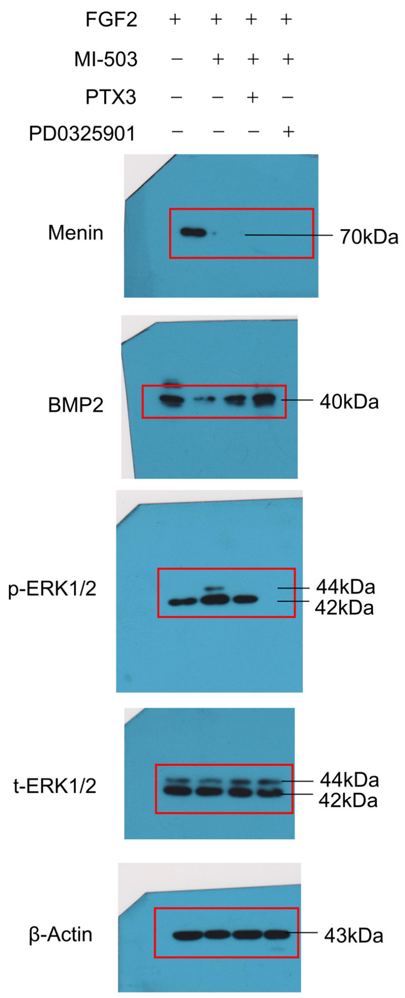

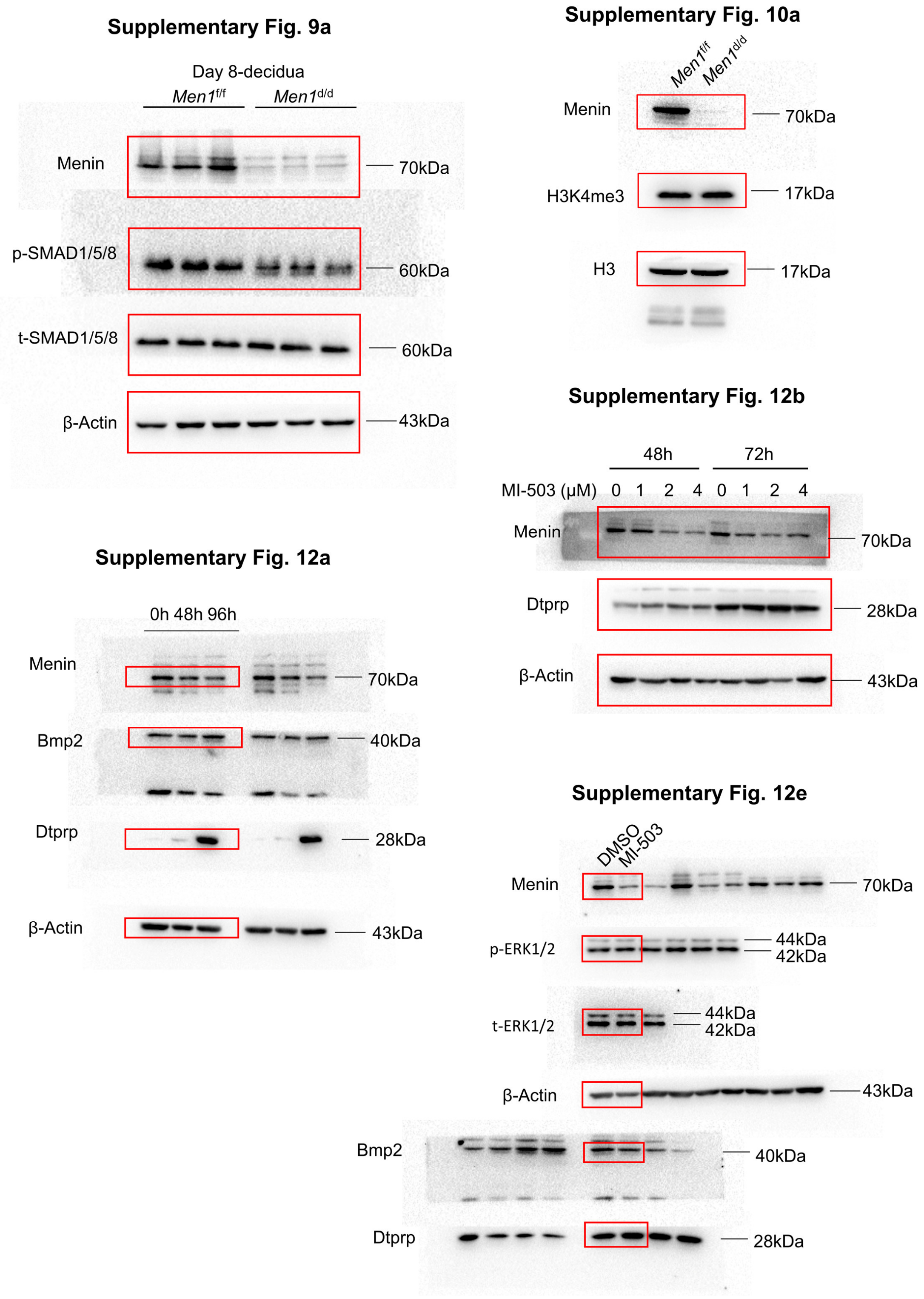

Supplementary Fig. 13 Pictures of the uncropped blots.

Supplementary Table 1: Antibody information

**Antibodies**	**Company**	**Catalog No.**
Menin	Bethyl	A300-105A
β-Actin	Bioworld	AP0060
COX2	Santa Cruz	sc-1746
Ki67	Servicebio	GB121141
BrdU	Abcam	ab6326
OCT4	Cell Signaling Technology	2840
Dtprp	Homemade	N/A
β-Catenin	Abcam	ab6302
FOXA2	Abcam	ab108422
H3K27me3	Cell Signaling Technology	9733
PCNA	Santa Cruz	sc-7907
PL1	Santa Cruz	sc-34713
3β-HSD	Santa Cruz	sc-30820
P450-scc	Santa Cruz	sc-18043
PR	Cell Signaling Technology	8757
HAND2	Santa Cruz	sc-9409
p27	Abcam	ab32034
pH3	Cell Signaling Technology	9701
BMP2	Abcam	ab14933
p-SMAD1/5/8	Cell Signaling Technology	9516

t-SMAD1/5/8	Cell Signaling Technology	9743
p-ERK1/2	Cell Signaling Technology	4370
ERK1/2	Cell Signaling Technology	4695
H3K4me3	Abcam	ab8580
H3	Abmart	TD6932
Anti-Rabbit IgG	Cell Signaling Technology	3900
Anti-Digoxigenin-AP, Fab fragments	Roche	11093274910
Cy^{TM}3 AffiniPure Goat Anti-Rabbit IgG(H+L)	Jackson ImmunoResearch	111-165-144

Supplementary Table 2. Primer list.

Quantitative real time PCR primer

Gene	Forward primer (5'-3')	Reverse primer (5'-3')
Men1	cccactcaccctttatca	cggcagtagttgtagtct
Gapdh	tggcaaagtggagattgttgcc	aagatggtgatgggcttcccg
Bmp2	gatctgtaccgcaggcactca	agttcctccacggcttcttcg
Wnt4	aggagtgccaataccagt	gctgaagagatggcgtatac
Ptgs2	gtctggtgcctggtctgatgat	gtggtaaccgctcaggtgttg
Pgr	acctgatctaatcctaaatga	attgtgttaagaagtagtaagac
Hoxa10	ggcagttccaaaggcgaaaa	caaaaaaagccagaacaaac
Hand2	tcggttatctagtgctgtc	atacttacaatgtttacaccttca

Angpt1	ttcttcgctgccattctgactc	cgttggtgttgtactgctctg
Angpt2	acggtcaacaactcgctcct	ttccgcacagtctctgaaggt
Cdkn1b	tcaaacgtgagagtgtctaacg	ccgggccgaagagatttctg
Fgfr1	acctcaccgctctacctgga	tctggctatggaagtcgctctt
Fgf2	agaacggcggcttcttcct	tggcacacactcccttgataga
Id1	cgaggtggtacttggtctgt	ccttgaggcgtgagtagca
Ptx3	agcgtgcatcctgtgagacca	tcctttccacccaccaccaaca

ChIP assay primers

**Gene**	**Forward primer (5'-3')**	**Reverse primer (5'-3')**
Ptx3	aggacaagctgataacactt	catctgcgagttctccag
Sfrp2	gcaaggcaatcaccagag	ctggctgcgttgagaatg
Twist2	ttggtggatgggtatctgt	aagagcggattgtctgtac
Snx10	cttggcttgctgagtgtt	cctcacaactggtcaatatg
Adora2b	cggctatcaagactctcct	ggacgagcacttctcaac
