# Supplementary material for: Menin directs regionalized decidual transformation through epigenetically setting PTX3 to balance FGF and BMP signaling
Source: Nat Commun. 2022 Feb 22;13:1006. doi: 10.1038/s41467-022-28657-2 (PMC8864016; doi:10.1038/s41467-022-28657-2)
Supplement: Supplementary file 3 — Description of Additional Supplementary Files [file 41467_2022_28657_MOESM3_ESM.pdf]

Description of Additional Supplementary Files

File Name: Supplementary Data 1
Description: Genes significantly downregulated by Men1 ablation in day 8 uteri.

File Name: Supplementary Data 2
Description: Genes significantly upregulated by Men1 ablation in day 8 uteri.

File Name: Supplementary Data 3
Description: Gene Ontology (GO) analysis for genes downregulated by Men1 ablation as reported by DAVID.

File Name: Supplementary Data 4
Description: Gene Ontology (GO) analysis for genes upregulated by Men1 ablation as reported by DAVID.

File Name: Supplementary Data 5
Description: Lists of genes for Cytokinesis/response to FGF/ response to BMP.
