# Supplementary material for: Menin directs regionalized decidual transformation through epigenetically setting PTX3 to balance FGF and BMP signaling
Source: Nat Commun. 2022 Feb 22;13:1006. doi: 10.1038/s41467-022-28657-2 (PMC8864016; doi:10.1038/s41467-022-28657-2)
Supplement: Supplementary file 9 — Reporting Summary [file 41467_2022_28657_MOESM9_ESM.pdf]

nature portfolio

Corresponding author(s): Haibin Wang

Last updated by author(s): Jan 24, 2022

Reporting Summary

Nature Portfolio wishes to improve the reproducibility of the work that we publish. This form provides structure for consistency and transparency in reporting. For further information on Nature Portfolio policies, see our Editorial Policies and the Editorial Policy Checklist.

Statistics

For all statistical analyses, confirm that the following items are present in the figure legend, table legend, main text, or Methods section.

n/a	Confirmed	
☐	☒	The exact sample size (*n*) for each experimental group/condition, given as a discrete number and unit of measurement
☐	☒	A statement on whether measurements were taken from distinct samples or whether the same sample was measured repeatedly
☐	☒	The statistical test(s) used AND whether they are one- or two-sided<br>*Only common tests should be described solely by name; describe more complex techniques in the Methods section.*
☒	☐	A description of all covariates tested
☐	☒	A description of any assumptions or corrections, such as tests of normality and adjustment for multiple comparisons
☐	☒	A full description of the statistical parameters including central tendency (e.g. means) or other basic estimates (e.g. regression coefficient) AND variation (e.g. standard deviation) or associated estimates of uncertainty (e.g. confidence intervals)
☐	☒	For null hypothesis testing, the test statistic (e.g. *F*, *t*, *r*) with confidence intervals, effect sizes, degrees of freedom and *P* value noted<br>*Give P values as exact values whenever suitable.*
☒	☐	For Bayesian analysis, information on the choice of priors and Markov chain Monte Carlo settings
☒	☐	For hierarchical and complex designs, identification of the appropriate level for tests and full reporting of outcomes
☐	☒	Estimates of effect sizes (e.g. Cohen's *d*, Pearson's *r*), indicating how they were calculated

Our web collection on statistics for biologists contains articles on many of the points above.

Software and code

Policy information about availability of computer code

Data collection	Leica DM2500 light microscope for image capturing<br>QuantStudio5 for Real-Time PCR data collection<br>ChemiDocTMXRS+ (BIO-RAD) for western blot<br>Beckman CytoFlex for Flow cytometry data collection
Data analysis	FlowJo_v10 software was used for Flow cytometry data analysis;<br>Trimgalore were used to filter raw sequencing data;<br>STAR 2.7.3a for aligment;<br>EdgeR 3.9 package in R was used for FPRM calculation;<br>MACS2 2.2.7.1 was used for peakcall of ChIP-Seq data;<br>ggplot2 3.3.5 was used for visualization;<br>GSEA 4.1 was used for pathway enrichment;<br>Complexheatmap 2.4.3 in R was used for heatmap generation;<br>ChIPseeker 1.24.0 was used for peak annotation;<br>ngs.plot.r 2.61 was used for generate peak heatmap of ChIP-Seq;<br>IGV 2.5.2 was used for peak visulization;<br>ChIPseqSpikeInFree 1.2.4 was used for normalization.

For manuscripts utilizing custom algorithms or software that are central to the research but not yet described in published literature, software must be made available to editors and reviewers. We strongly encourage code deposition in a community repository (e.g. GitHub). See the Nature Portfolio guidelines for submitting code & software for further information.

March 2021

Data

Policy information about availability of data

All manuscripts must include a data availability statement. This statement should provide the following information, where applicable:

- Accession codes, unique identifiers, or web links for publicly available datasets
- A description of any restrictions on data availability
- For clinical datasets or third party data, please ensure that the statement adheres to our policy

All data supporting the findings of this study are available within the paper and its supplementary information files.

Field-specific reporting

Please select the one below that is the best fit for your research. If you are not sure, read the appropriate sections before making your selection.

[x] Life sciences [ ] Behavioural & social sciences [ ] Ecological, evolutionary & environmental sciences

For a reference copy of the document with all sections, see nature.com/documents/nr-reporting-summary-flat.pdf

Life sciences study design

All studies must disclose on these points even when the disclosure is negative.

Sample size	For animal work, at least 3 independent mice were used for each experimental group. For quantitative real-time PCR, we performed 3 biological replicates to be able to perform statistical analysis. The sample sizes of high-throughput sequencing were based on previously published openly shared datasets found in various sources. For in vitro experiments, sample sizes were determined based on our pilot experiments.
Data exclusions	No data were excluded.
Replication	All in vivo and in vitro functional and phenotypic experiments were performed in at least biological triplicate to ensure reproducibility. Similar results were obtained in at least three independent experiments.
Randomization	All samples were allocated at random.
Blinding	We were blinded to group allocation during data collection.

Reporting for specific materials, systems and methods

We require information from authors about some types of materials, experimental systems and methods used in many studies. Here, indicate whether each material, system or method listed is relevant to your study. If you are not sure if a list item applies to your research, read the appropriate section before selecting a response.

Materials & experimental systems

n/a	Involved in the study	
[ ]	[x]	Antibodies
[x]	[ ]	Eukaryotic cell lines
[x]	[ ]	Palaeontology and archaeology
[ ]	[x]	Animals and other organisms
[x]	[ ]	Human research participants
[x]	[ ]	Clinical data
[x]	[ ]	Dual use research of concern

Methods

n/a	Involved in the study	
[ ]	[x]	ChIP-seq
[ ]	[x]	Flow cytometry
[x]	[ ]	MRI-based neuroimaging

Antibodies

Antibodies used	For immunostaining, following antibodies were used: Menin (A300-105A, Bethyl), COX2 (sc-1746, Santa Cruz), Dtprp (Homemade), Ki-67 (GB121141, Servicebio), PL1(P-17) (sc-34713, Santa Cruz), 3β-HSD (sc-30820, Santa Cruz), p450scc (sc-18043, Santa Cruz), PR (D8Q2J) (8757, Cell Signaling Technology), HAND2 (sc-9409, Santa Cruz), p27(Y236)(ab32034, Abcam), pH3 (9701, Cell Signaling Technology), ERK1/2(137F5) (4695, Cell Signaling Technology), p-ERK1/2(D13.14.4E) (4370, Cell Signaling Technology), OCT4(C30A3) (2840, Cell Signaling Technology), β-Catenin (ab6302, Abcam), H3K27me3 (C36B11)(9733, Cell Signaling Technology), BrdU (BU1/75) (ab6326, Abcam), PCNA (sc-7907, Santa Cruz), Foxa2(EPR4466) (ab108422, Abcam), Anti-Digoxigenin-AP, Fab fragments (1093274910, Roche) and Cy™3 AffiniPure Goat Anti-Rabbit IgG (H+L) (111-165-144, Jackson ImmunoResearch).<br>For Western blotting, the following antibodies were used: Menin (A300-105A, Bethyl), β-Actin (AP0060, Bioworld), Dtprp (homemade), ERK1/2 (137F5)(4695, Cell Signaling Technology), p-ERK1/2 (D13.14.4E)(4370, Cell Signaling Technology), BMP2

(ab32034, Abcam), SMAD1/5/8 (9743, Cell Signaling Technology), p-SMAD1/5/8(41D10) (9516, Cell Signaling Technology), H3K4me3 (ab8580, Abcam) and H3 (TD6932, Abmart).
For ChIP-seq and ChIP-qPCR, the following antibodies were used: Menin (A300-105A, Bethyl), H3K4me3 (ab8580, Abcam) and Rabbit IgG(DA1E) (3900, Cell Signaling Technology).

Validation

Our newly developed antibody against Dtprp was extensively tested for specificity. See Figure 3e and f and Supplementary Figure 3d. All the other antibodies used have been extensively utilized in the literature and have been validated previously. All antibodies were commercially available. Listed below are information available.
Menin (A300-105A, Bethyl): https://www.bethyl.com/product/A300-105A
COX2 (sc-1746, Santa Cruz): https://www.scbt.com/p/cox-2-antibody-n-20
Ki-67 (GB121141, Servicebio): https://www.servicebio.cn/goodsdetail?id=6801
PL1 (sc-34713, Santa Cruz): https://www.scbt.com/p/placental-lactogen-i-antibody-p-17
3β-HSD (sc-30820, Santa Cruz): https://www.scbt.com/p/3beta-hsd-antibody-p-18
PR (8757, Cell Signaling Technology): https://www.cellsignal.cn/products/primary-antibodies/progesterone-receptor-a-b-d8q2j-xp-rabbit-mab/8757
HAND2 (sc-9409, Santa Cruz): https://www.scbt.com/p/dhand-antibody-m-19
p27(ab32034, Abcam): https://www.abcam.cn/p27-kip-1-antibody-y236-ab32034.html
pH3 (9701, Cell Signaling Technology): https://www.cellsignal.cn/products/primary-antibodies/phospho-histone-h3-ser10-antibody/9701
ERK1/2 (4695, Cell Signaling Technology): https://www.cellsignal.cn/products/primary-antibodies/p44-42-mapk-erk1-2-137f5-rabbit-mab/4695
p-ERK1/2 (4370, Cell Signaling Technology): https://www.cellsignal.cn/products/primary-antibodies/phospho-p44-42-mapk-erk1-2-thr202-tyr204-d13-14-4e-xp-rabbit-mab/4370
OCT4 (2840, Cell Signaling Technology):
β-Catenin (ab6302, Abcam): https://www.abcam.cn/beta-catenin-antibody-ab6302.html
H3K27me3 (9733, Cell Signaling Technology): https://www.cellsignal.cn/products/primary-antibodies/tri-methyl-histone-h3-lys27-c36b11-rabbit-mab/9733
BrdU (ab6326, Abcam): https://www.abcam.cn/brdu-antibody-bu175-icr1-proliferation-marker-ab6326.html
PCNA (sc-7907, Santa Cruz): https://www.scbt.com/p/pcna-antibody-fl-261
Foxa2 (ab108422, Abcam): https://www.abcam.cn/foxa2-antibody-epr4466-ab108422.html
β-Actin (AP0060, Bioworld): https://www.bioworlde.com/Primary-Antibodies/25097.html
BMP2 (ab14933, Abcam): https://www.abcam.cn/bmp2-antibody-ab14933.html
SMAD1/5/8 (9743, Cell Signaling Technology): https://www.cellsignal.cn/products/primary-antibodies/smad1-antibody/9743
p-SMAD1/5/8 (9516, Cell Signaling Technology): https://www.cellsignal.cn/products/primary-antibodies/phospho-smad1-5-ser463-465-41d10-rabbit-mab/9516
H3K4me3 (ab8580, Abcam): https://www.abcam.cn/histone-h3-tri-methyl-k4-antibody-chip-grade-ab8580.html
H3 (TD6932, Abmart): http://www.ab-mart.com.cn/page.aspx?node=%2065%20&id=%2031215
Rabbit IgG (3900, Cell Signaling Technology): https://www.cellsignal.cn/products/primary-antibodies/rabbit-da1e-mab-igg-xp-isotype-control/3900
Anti-Digoxigenin-AP, Fab fragments (1093274910, Roche) https://www.sigmaaldrich.cn/CN/zh/product/roche/11093274910
Relevant citation for p450scc (sc-18043, Santa Cruz): J Clin Invest. 2018 Jan 2;128(1):175-189.

Animals and other organisms

Policy information about studies involving animals; ARRIVE guidelines recommended for reporting animal research

Laboratory animals

2-month-old C57BL/6 male and female mice were used in the present study. Mice were housed in the animal care facility of Xiamen University with controlled environment (22±2°C, 50 -60% humidity, 12-h light/dark cycle, lights on at 7AM) and free access to food and water according to the guidelines for the care and use of laboratory animals.

Wild animals

No wild animals were used.

Field-collected samples

No field collected sample were used.

Ethics oversight

Mice were housed in the animal care facility of Xiamen University according to the guidelines for the care and use of laboratory animals. All experimental procedures were approved by the Animal Welfare Committee of Research Organization (X200811), Xiamen University.

Note that full information on the approval of the study protocol must also be provided in the manuscript.

ChIP-seq

Data deposition

- [x] Confirm that both raw and final processed data have been deposited in a public database such as GEO.
- [x] Confirm that you have deposited or provided access to graph files (e.g. BED files) for the called peaks.

Data access links<br>*May remain private before publication.*	GSE182539 for ChIP-Seq data;<br>GSE191327 for ChIP-Seq data;<br>GSE182525 for RNA-Seq data.
Files in database submission	GSM5530886 D8_WT_1<br>GSM5530887 D8_WT_2<br>GSM5530888 D8_Menin_KO_1<br>GSM5530889 D8_Menin_KO_2<br><br>GSM5531080 Menin ChIP_Seq in decidualized stromal cells repeat 1<br>GSM5531081 Menin ChIP_Seq in decidualized stromal cells repeat 2<br>GSM5531082 H3K4me3 ChIP_Seq in WT decidualized stromal cells repeat 1<br>GSM5531083 H3K4me3 ChIP_Seq in KO decidualized stromal cells repeat 1<br>GSM5743703 H3K4me3 ChIP_Seq in WT decidualized stromal cells repeat 2<br>GSM5743704 H3K4me3 ChIP_Seq in KO decidualized stromal cells repeat 2<br>GSM5531084 Input decidualized stromal cells
Genome browser session<br>(e.g. UCSC)	no longer applicable

Methodology

Replicates	Each experiment was repeat twice.
Sequencing depth	All of the data is Pair-End with 150bp of each read.<br>Menin re1: 48156925 unique mapped in total 54426277 reads<br>Menin re2:48801826 unique mapped in total 57396697 reads<br>H3K4me3 in WT rep1: 48801826 unique mapped in total 61957927 reads<br>H3K4me3 in WT rep2: 32273108 unique mapped in total 37354173 reads<br>H3K4me3 in KO rep1: 38918215 unique mapped in total 49233334 reads<br>H3K4me3 in KO rep2: 50573808 unique mapped in total 59263269 reads
Antibodies	Menin (A300-105A, Bethyl),H3K4me3 (ab8580, Abcam)
Peak calling parameters	macs2 callpeak -f BAMPE --nomodel -B with other default parameters
Data quality	There are 6101 and 8564 filtered peaks for Men1.<br>There are 29040 and 25229 filtered peaks for H3K4me3 in WT cells.<br>There are 24331 and 20463 filtered peaks for H3K4me3 in KO cells.
Software	STAR 2.7.3a for aligment; EdgeR 3.9 package in R was used for calculation of different peaks;<br>MACS2 2.2.7.1 was used for peakcall of ChIP-Seq data;<br>ggplot2 3.3.5 was used for visualization;<br>ChIPseeker 1.24.0 was used for peak annotation;<br>ngs.plot.r 2.61 was used for generate peak heatmap of ChIP-Seq;<br>IGV 2.5.2 was used for peak visualization.

Flow Cytometry

Plots

Confirm that:

- [x] The axis labels state the marker and fluorochrome used (e.g. CD4-FITC).
- [x] The axis scales are clearly visible. Include numbers along axes only for bottom left plot of group (a 'group' is an analysis of identical markers).
- [x] All plots are contour plots with outliers or pseudocolor plots.
- [x] A numerical value for number of cells or percentage (with statistics) is provided.

Methodology

Sample preparation	Day 8 decidual cells were digested and harvested. The cell pellet was suspended in 0.25 ml PBS after centrifugation; 1 ml of cold 80% ethanol was added dropwise under constant and gentle vortexing. Samples were incubated for 30 min on ice and subsequently overnight at -20°C before subjected to staining. Cell sediments were suspended in staining solution (PBS containing 5mg/ml Propidium Iodide and 2 mg/ml DNase-free RNase A) the next day.Samples were incubated for 30 min at 37°C in the dark. They were then subjected to flow cytometry.
Instrument	Beckman CytoFlex

Software	The FlowJo_v10 Software was used for FACS data analysis.
Cell population abundance	N/A
Gating strategy	SSC-A and FSC-A were used for loose gate. FSC-A and FSC-H were used to select single cells. The polyploid cells are distinguished by intensity value of FSC-A which is proportional to cell size and the intensity value of Propidium Iodide staining which is used to measure DNA content.

☒ Tick this box to confirm that a figure exemplifying the gating strategy is provided in the Supplementary Information.
